# Community-driven partnerships with Community-Engaged Research teams bring resources and reliable information to Baltimore residents

**DOI:** 10.1017/cts.2024.606

**Published:** 2024-10-23

**Authors:** Emily Kumpf, Veena Thamilselvan, Ethan Wang, Patricia Barger, Janice Gentry, Chevelle Bash, Donald Young, Samuel Byiringiro, Joann Bodurtha, Antoinette Brown, Minli Guo, Audrey Carter, Latrice Price, Percy Smith, Cyd Lacanienta, Cheryl Himmelfarb, Albert W. Wu

**Affiliations:** 1 Baltimore CONNECT, Baltimore, MD, USA; 2 Department of Health Policy and Management, Johns Hopkins Bloomberg School of Public Health, Baltimore, MD, USA; 3 Department of International Health, Johns Hopkins Bloomberg School of Public Health, Baltimore, MD, USA; 4 Kreiger School of Arts and Sciences, Johns Hopkins University, Baltimore, MD, USA; 5 The Family Tree, Baltimore, MD, USA; 6 Biddle Neighborhood Association, Baltimore, MD, USA; 7 Green and Health Homes Initiative, Baltimore, MD, USA; 8 Community Engagement Alliance for DC, Maryland and Virginia (CEAL DMV), Johns Hopkins School of Nursing, Baltimore, MD, USA; 9 Institute for Clinical Translational Research, Johns Hopkins School of Medicine, Baltimore, MD, USA; 10 Family History Tutorial, Johns Hopkins School of Medicine, Baltimore, MD, USA; 11 Our Daily Bread Employment Center, Catholic Charities, Baltimore, MD, USA; 12 Marketing Analytics, Johns Hopkins University Carey Business School, Baltimore, MD, USA; 13 Youth Ventures, Baltimore, MD, USA; 14 Oliver Community Association, Baltimore, MD, USA; 15 Infinite Legacy, Baltimore, MD, USA; 16 Nuwave Health Services, Baltimore, MD, USA

**Keywords:** Community engagement, DEI, community-academic partnership, diversity, equity, inclusion

## Abstract

This case study presents an analysis of community-driven partnerships, focusing on the nonprofit Baltimore CONNECT (BC) network and its collaborative efforts with a Community-Engaged Research (CEnR) team of the Johns Hopkins Institute for Clinical and Translational Research (ICTR). BC has built a network of over 30 community-based organizations to provide health and social services in Baltimore City. The study emphasizes the role of CEnR in supporting community-led decision-making, specifically in the planning and implementation of community health resource fairs. These fairs address social determinants of health by offering a variety of services, including health education, screenings, vaccinations, and resource distribution. The paper details the methods, resource mobilization, and collaborative framing processes in the execution of these fairs in a community-academic collaboration with the ICTR. Results from a 2.5-year period show the positive impact of the fairs on individuals, families, and the community at large in East Baltimore. The findings underscore the importance of community-led collaborations in addressing health disparities and improving overall community well-being. It concludes by reflecting on the sustained engagement, trust-building, and shared learning that emerges from such partnerships, suggesting a model for future community-academic health initiatives.

## Introduction

Community health resource fairs can be effective in increasing access to healthcare services in low-resourced communities through providing health education information, screening, vaccinations, healthcare insurance, and community resource [1,2]. Community resource fairs conducted within community-academic partnerships (CAP) have the potential to address social determinants of health (SDoH) and health disparities [3,4]. Less is known about the effectiveness of CAP resource fairs that utilize principles of Community-Engaged Research (CEnR) to implement community-driven processes to disseminate research findings and address health inequalities.

Investigators utilizing CEnR work in long-term, trusted relationships with individuals and organizations in communities, advancing bi-directional, mutually respectful, and beneficial partnerships [5]. CEnR teams can support programing of community-centered outreach in ways that leverage community strengths and resources. They can help to ensure that trusted scientific results being disseminated are responsive to community needs and that materials are culturally appropriate [6,7]. The Baltimore CONNECT network has shown how active community partners can drive CAP processes consistent with the theoretical frameworks of (1) CEnR and (2) social movement theory [8]. Community engagement is defined as having community members involved in the process of research [9]. In community engagement, involvement can occur at all stages of the research process and centers around mutual trust [9]. By having a community-engaged lens to research, there is potential to reduce health disparities [9]. Social movement theory describes organizations and individuals as change agents, who are intrinsically motivated to drive change in a community [10].

Community members share in decision-making and drive actions taken to enact positive changes in a community. This is consistent with the Patient-Centered Outcomes Research Institute (PCORI) engagement rubric and Environmental Protection Agency public participation guide [11,12]. The PCORI rubric shows opportunities for bi-directional engagement between community stakeholders and investigators throughout the research cycle. The Public Participation Guide framework can also be utilized in the development and implementation of community interventions. Public participation is rooted in seven core values and the right of the public to be involved in decision-making processes [13]. All of those involved in the outcomes of a decision participate in a way that is meaningful and fosters sustainability.

## Baltimore CONNECT

The CAP was led by Baltimore CONNECT, Inc. (BC), a 501c(3) nonprofit organization committed to strengthening a membership network of community-based organizations (CBOs) providing safety net services to communities in Baltimore City [14]. BC started in 2013 with 20 CBO members serving East Baltimore. Today, BC’s member organizations have over 35 CBOs, faith-based organizations, and neighborhood association members, and partners with community leaders, health professionals, and trainees advocating for and connecting residents to coordinated health care and human services. Serving as a bridge between hospitals and surrounding neighborhoods, BC’s vision is collaboration to “achieve optimum health and well-being for all.” The goal of BC and its safety net provider network is to reach underserved communities with limited access to resources, foster collaboration and coordination among local organizations to de-silo resources, reduce duplication of efforts, and help clients access needed services. The strength of BC is the collective reach of its membership throughout Baltimore City, allowing the group to represent residents’ values and needs, and build service capacity through partnerships. Additionally, its connections with the health system have allowed BC to connect with academic trainees to volunteer and support logistics. BC provides opportunities for undergraduate, graduate, and post-graduate trainees with opportunities for CEnR practices and experiences. Baltimore CONNECT, inc. is governed by a Board of Directors of seventeen members, including both leaders at academic medical institutions and community leaders. BC has obtained grant funding to support its growth and become a nonprofit organization with a full-time executive director. At the start of the partnership with Institute for Clinical and Translational Research (ICTR) and Community Engagement Alliance (CEAL) in 2020, member organizations reported serving over 43,000 individuals in Baltimore City, most of whom are members of historically underserved groups (shown in Table 1). Since then, BC members have met weekly on an hour-long Zoom meeting to exchange information.

**Table 1.** Baltimore connect members’ community reach (2021 assessment)

Name of organization	# Unique Clients	# African American	% African American	# Latinx	% Latinx	# ≥ 65 years	% ≥ 65 years	# Unstable Home Address	% Unstable Home Address	# Substance abuse	% Substance Abuse	# Recently Released	% Recently Released	# Willing to Get a COVID Test	% Willing to Get a COVID Test
Action in Maturity	3200	2800	88%	95	3%	3200	100%							640	20%
Asylee Women Enterprise	1000	300	30%	700	70%	50	5%	900	90%					1000	100%
Banner Neighborhoods Community Corporation	400	300	75%	25	6%	200	50%							280	70%
Bea Gaddy Family Center	7250	5800	80%	725	10%	5075	70%	4713	65%	4713	65%	120	2%	4350	60%
Civic Works	3000	918	31%	390	13%	963	32%					1650	55%	2100	70%
Dayspring Programs, Inc.	164	100	61%	10	6%	0	0%	0	0%					82	50%
Echo Resource Development, Inc.	3500	2500	71%	750	21%	1000	29%	250	7%	1000	29%	1000	29%	2450	70%
Esperanza Center	8500	850	10%	7000	82%	1500	18%							4250	50%
Fort Worthington Neighborhood Association	60	60	100%	0	0%	20	33%	3	5%	4	7%	4	7%	36	60%
Franciscan Center	3000	2400	80%	65	2%	1000	33%	1000	33%	2500	83%	125	4%	1500	50%
Green and Healthy Homes Initiatives	538	363	67%	13	2%	100	19%							108	20%
Helping Up Mission	964	320	33%	13	1%	34	4%	964	100%	884	92%			964	100%
Manna House	3000	2700	90%	90	3%	450	15%	3000	100%	2250	75%	450	15%	1200	40%
Maryland New Directions	196	192	98%	10	5%	2	1%	78	40%	49	25%	4	2%	98	50%
McElderry Park Community Association	400	360	90%	40	10%	110	28%	360	90%	220	55%	220	55%	80	20%
Men and Families Center, Inc.	3100	2800	90%	155	5%	2170	70%	1400	45%	160	5%	420	14%	2170	70%
Moveable Feast	1575	1019	65%	14	1%	569	36%							788	50%
Roberta’s House	1800	1700	94%	10	1%	720	40%	50	3%					900	50%
St. Ambrose Housing Aid Center	40	38	95%	0	0%	0	0%	40	100%	12	30%	8	20%	16	40%
The Door	190	152	80%	43	23%	150	79%							95	50%
Zion Baptist Church	760	744	98%	14	2%	456	60%			45	6%			152	20%
Total	42,637	26,416		10,162		17,769		12,758		11,837		4,001		23,258	

## Johns Hopkins ICTR community and collaboration core (core)

The CenR team in this partnership was the Community and Collaboration Core (Core) of the Johns Hopkins ICTR and the National Institutes of Health (NIH) CEAL program. Since 2007, the Core has promoted meaningful engagement of community partners across the research life cycle and the translational research phases through enhanced CenR training, joint planning of engagement activities, co-design of studies, and increased opportunities for stakeholder participation in the research [15,16]. The Core has developed shared-governance models with a diverse community of partners to support trustworthiness and shared learning of best practices, leading to increased public involvement and support for research; improved health in Maryland and the nation; and a training pipeline for the next generation of CenR scientists [16,17]. In 2020, ICTR and the School of Nursing’s CEAL program had already begun working with community grassroots organizations to develop outreach and engagement approaches that disseminate trustworthy, science-based information to communities about COVID-19 in the midst of “the greatest global public health crisis in more than a century” [18].

## Resource fair collaborative team (dream team)

The *Dream Team (DT)* is a community-driven subcommittee of BC composed of CBO members and CEnR researchers from an academic institution. DT was convened in 2020 as a small workgroup of 7 members from different CBOs resource priorities in Baltimore. DT members worked together to bring resources to Baltimore residents through the planning and implementation of bi-annual community resource fairs.

The committee strengthened partnerships for the resource fairs by leveraging the collective talents and resources of BC members, by engaging multiple CBOs working with diverse populations (e.g., older adults, youth, individuals experiencing homelessness, chemical dependency, LGBTQIA+, persons living in recovery, LatinX, returning citizens, undocumented people). DT anticipated equity challenges in planning and strategized solutions to promote inclusion. An overarching theme echoed in implementation design was the importance of inclusivity, respect, and compromise.

*“At the end of the day, we are partners, and we want to support each other.” Co-chair, DT Committee*

This paper presents a case study demonstrating how community partners can drive planning and processes with a CEnR team that supports community-centered decision-making. This enables the implementation of community health resource fairs as an effective platform for disseminating health information and resources. This intervention addresses SDoH at multiple levels of the socioecological model, aiming to positively impact individuals, families, communities, and organizations in Baltimore.

## Methods

This case study examines a community-led partnership between a CBO network and the ICTR CenR team. The study covers 2.5 years from the spring of 2021 through the fall of 2023. It includes the planning and implementation of five community-driven resource fairs across Baltimore City and the supportive capacity-building role played by the ICTR CenR team. The case can be viewed through a conceptual framework built on social movement theory and CenR and includes problem identification through a needs assessment, a collaborative framing process, and collective action to achieve community and system changes [8].

### CBO needs assessment

In March 2020, BC began holding weekly online meetings in response to community needs stemming from the COVID-19 pandemic. Members voiced the need to make Baltimore City residents aware of the CBO services locally available. Meeting time was allotted for CBO representatives to share clients’ service needs and provide information about accessible resources which was shared with the ICTR team. Among these was the need for reliable information about the SARS-CoV-2 to counter growing misinformation about COVID-19 testing, treatment, and vaccines. BC’s 2021 assessment of client reach within its network revealed members’ significant coverage of the communities impacted by the pandemic as well as minoritized groups’ willingness to take the COVID-19 test (see Table 1).

BC conducted an ongoing community needs assessment in the weekly Zoom meetings to understand the evolving community needs and preferences in real time. In response, CBO members convened the DT and identified the intervention community resource fairs, as an effective way to meet these needs simultaneously.

### Framing process

Through weekly virtual meetings, planning was self-directed by members of the committee, who were assigned specific roles and responsibilities based on expertise and interests. The members decided where, when, and how resource fairs would occur. This included identifying sites, food and music vendors, service providers, speakers, and dignitaries, as well as controlling the event budget. Decision-making was made by consensus, discussed at weekly meetings, and documented on the shared online spreadsheet. Team decisions included which dignitaries to invite, vendors to engage, and what activities to fund. General administrative support was provided by BC staff who helped manage registration, update social media for advertising, manage payments to vendors, and liaise between the steering committee and planning committees. The goal was for this committee to be community-driven, to ensure members working closest to the community had ownership over the development and execution process.

**Figure 1.**
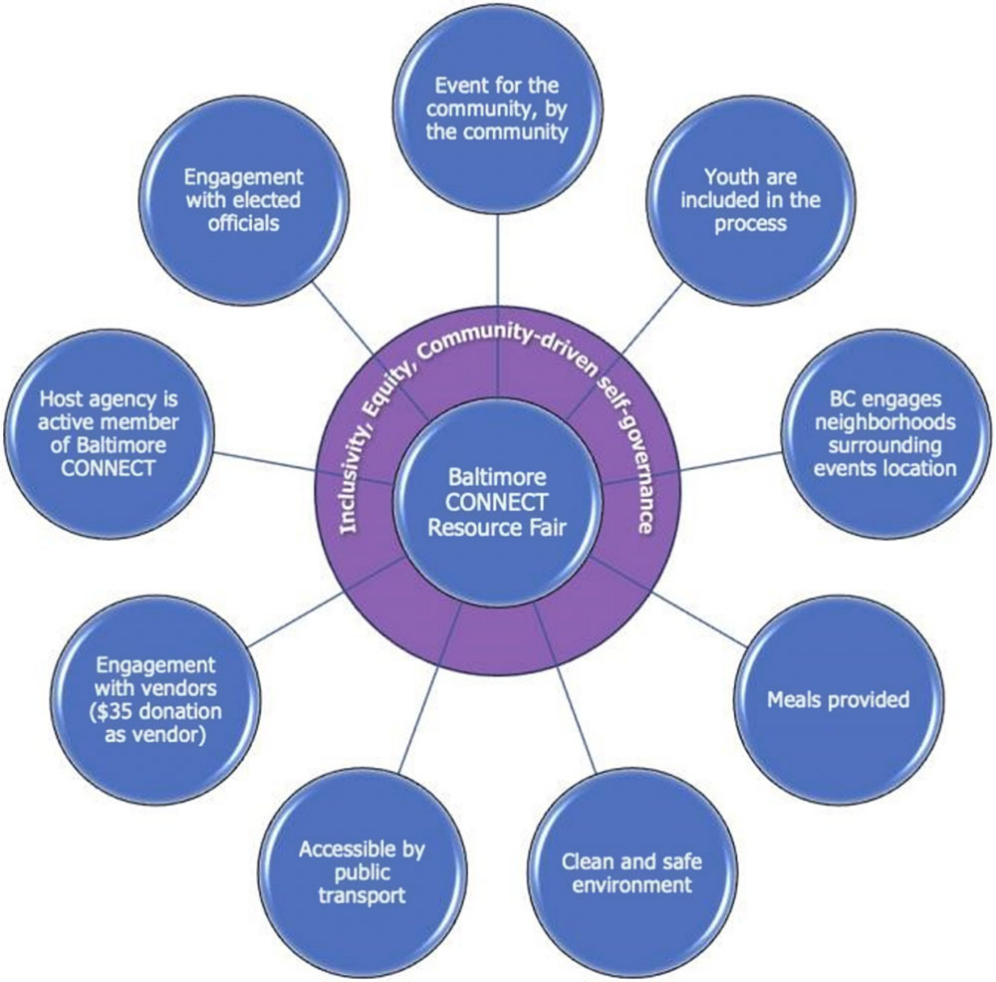
Principles identified by the dream team.

The DT developed operating principles for BC’s brand of community engagement and outreach for its resource fairs: (1) The resource fair is inclusive; for the community, by the community, striving to reach all segments of the community. (2) BC engages neighborhoods surrounding the event’s location. (3) The host agency of the event is an active member of BC. (4) Vendors are engaged throughout the process. (5) The location is accessible by public transportation. (6) The environment is clean and safe. (7) Food is provided. (8) Youth are included in the process through volunteering and receiving stipends. (9) There is engagement with elected officials (shown in Figure 1).

### Collective action

**Figure 2.**
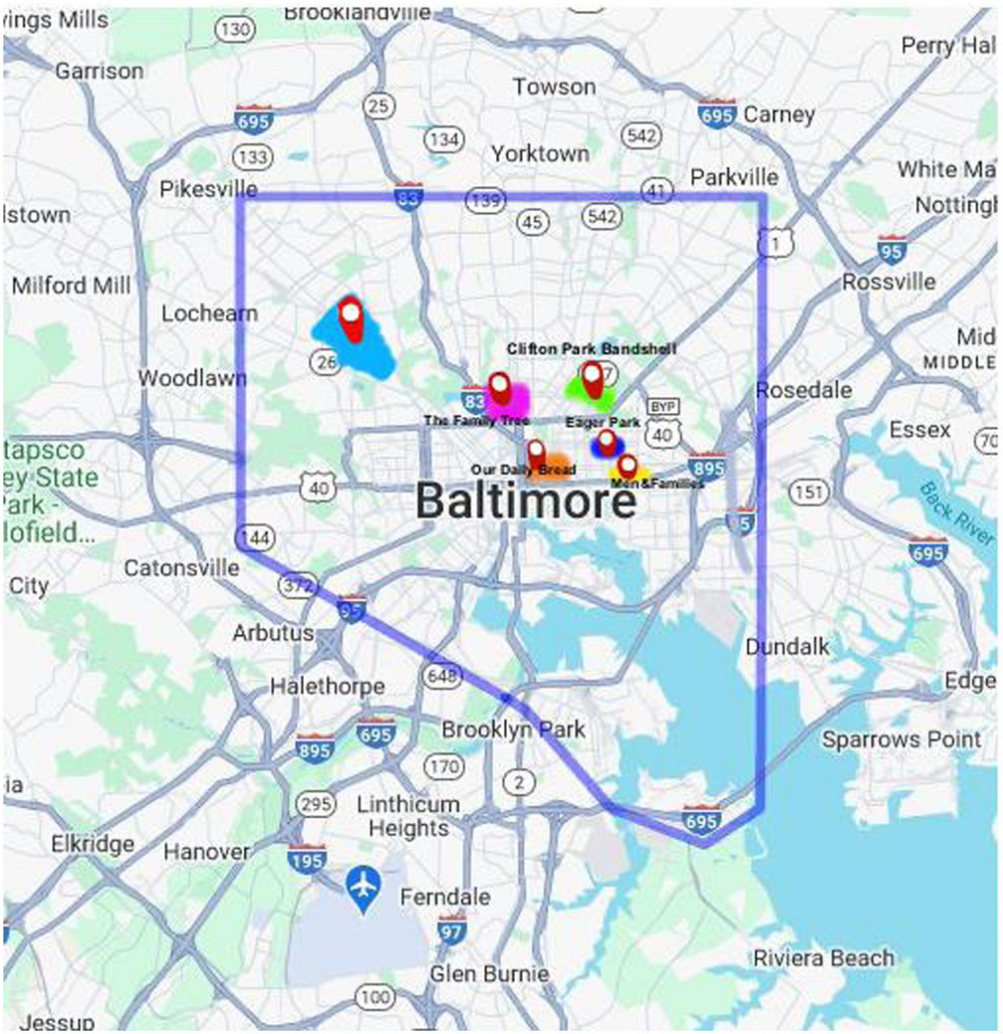
Distribution of resource fairs in Baltimore city. This map shows the movement of the resource fair to different underserved neighborhoods in Baltimore City with the aim of spreading the reach of the resource fair.

Community engagement in implementation was instrumental in the mobilization and planning of the resource fairs. The co-chair of the DT emphasized: “Through trial and error, I’ve found it to be critical and utmostly respectful to engage the community at all levels of planning.” The community was at the center of driving the planning and the decision-making processes. This included canvassing, reaching out to the city for permits, identifying local association leaders, ensuring youth voices were heard, and familiarizing the team with the neighborhood and local school. Canvassing and building relationships with local community leaders occurred in the following five areas where the resource fairs took place: (1) Eager Park (2) Clifton Park (3) Our Daily Bread Employment Center (zipcode: 21,202) (4) The Family Tree (zipcode: 21,218) (5) Men and Families Center (zipcode: 21,205). The map in Figure 2 depicts the areas where canvassing and outreach occurred around each resource fair.

The DT developed detailed and transparent action plans with timelines, tasks, and responsible parties. This included strategies for community engagement, outreach, and communication. The administrative operationalization used cloud-based live documents to manage fairs, specifically with a shared planning spreadsheet, an event registration form on Eventbrite, and the official BC website. The spreadsheet was used to keep track of the timeline of activities, action items for each committee member, and running budget. Since this was a live document that everyone could access and edit, there was clarity in how the action items were divided, transparency on how the budget was allocated, and accessibility via smartphone for members with limited technical skills. The BC website, which is one of the main methods for people and vendors to see updated events and community opportunities, was used to increase awareness within the local community and to register vendors for the fair.

To track the number of attendees who visited the overall event, and each vendor table, the team distributed clickers to each vendor and at the main registration table and asked them to track the number of visitors. We evaluated qualitative data and data collected at five resource fairs over 2.5 years for a total of 83 vendor organizations.

At each resource fair, the check-in and check-out process was driven by BC staff and volunteers. When partner organizations arrived at the event, they were checked in at the vendor registration table. Vendors scanned a QR code on their smartphone, which directed them to a customized online check-in survey. This form captured information including name, contact details, and organizational affiliation. Tally counters (i.e., clickers) were given to each vendor to track the number of participants who stopped by their table. They also receive personal protective gear (hand sanitizers and facial masks) as needed.

**Figure 3.**
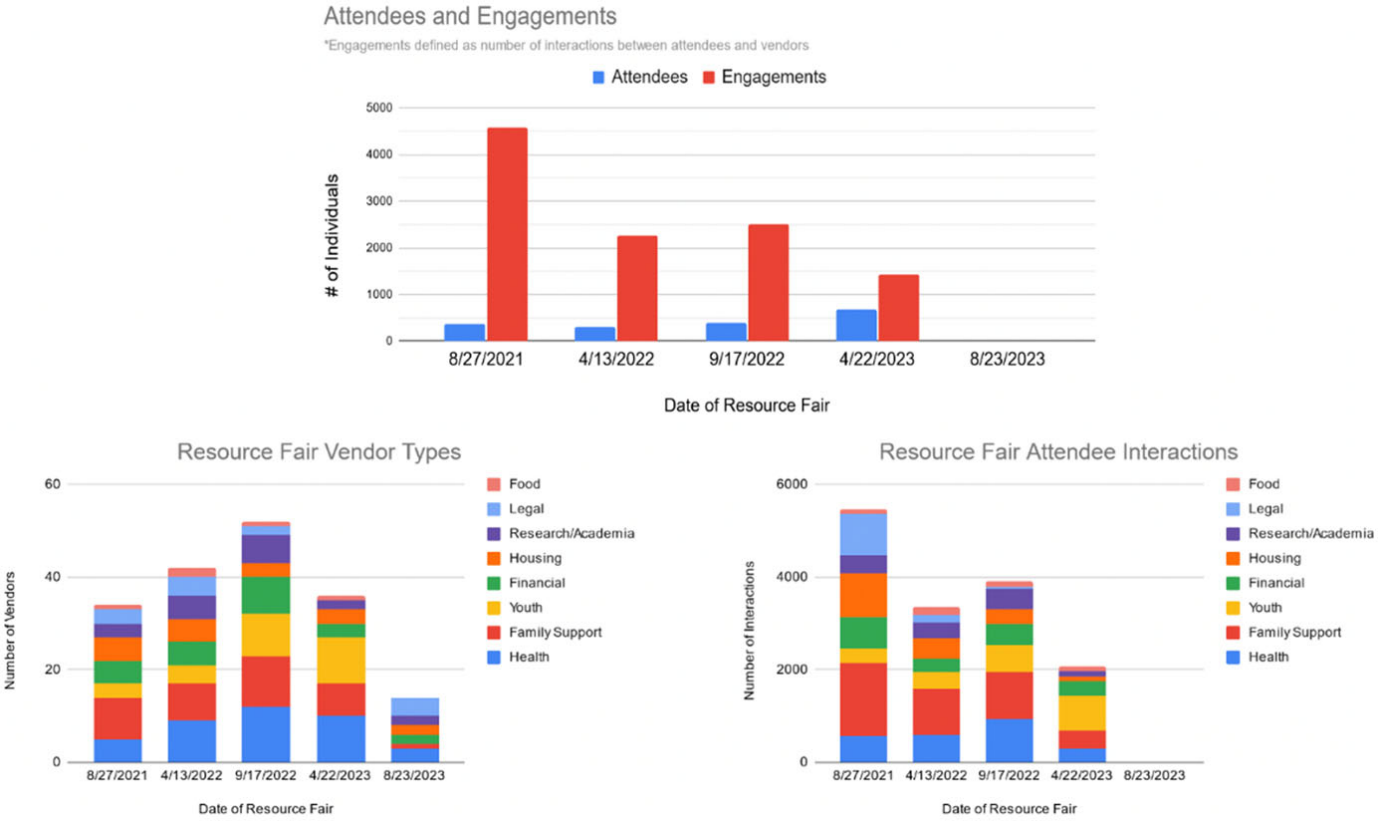
Summary of attendee engagements with vendors by service type each vendor had activities at their table that addressed SDoH (e.g., health, housing, legal, youth, food, financial, and family support) or their research and academic mission. Vendors may fit into more than one category. Categories were: health organizations that specifically addressed health and examples of activities include vaccine distribution, providing resources about COVID, organ donation, participation in research, etc.; housing which included providing housing for individuals experiencing homelessness and helping individuals and families find affordable housing; legal which provided services including help in finding insurance, filing court claims for various needs, and taxes; food security which included providing direct food transfers and connecting families to food banks; financial which provided access to tech resources that would have been otherwise difficult to access; family support which included vendors providing wraparound support for mothers and caregivers, and grief counseling; youth which focused services including school materials (books, backpacks, school supplies), information about housing, family support, and food security.

A total of 83 different vendors participated in the five resource fairs. Each vendor had different activities at each table that addressed SDoH. Figure 3 shows the number of vendors that focused on different SDoH (health, housing, legal, youth, food, financial, and family support), and those that had a research or research and academic mission.

Post-event, the organizing team encouraged vendors to submit information to an online check-out survey form. The survey measurement included questions that captured qualitative data around vendor’ overall experience of the event. The survey tool also recorded activities conducted by the exhibitor vendor, number of interactions, and the type and quantity of giveaways distributed. Throughout the implementation of the resource fairs, the team monitored progress of the action plans and communicated with the community vendors to gather feedback and adjust as needed. After each resource fair, the DT met to debrief and reflect on what worked well, as well as areas for improvement moving forward. The feedback from community vendors led to co-hosting with a community partner organization for resource fairs #3-5. In addition, the qualitative data and feedback from vendors led to the expansion beyond East Baltimore.

## Results

Overall, 75 CBOs and 9 research/ academic organizations were involved as vendors in the 5 resource fairs. Throughout the resource fairs, the number of attendees increased from 375 attendees in the first fair to 670 attendees in the fourth resource fair. The engagements with vendors decreased over the same period. The number of attendees and the engagement with vendors from the first four resource fairs are depicted in Figure 3. (data on engagements from the fifth resource fair was not captured because counters were not distributed to vendors at the event.)

Organizations providing family support resources and health resources received a high degree of engagement from attendees. Each resource fair had vendors from the following categories: health organizations, housing activities, legal services, food security vendors, financial services, family support services, youth-serving vendors, as well as academic and research fair tables. In response to community requests for more support for youth, there was an increasing amount of engagement from attendees visiting youth service-related vendors, with an increase in youth engagement from 5.7% in the first resource fair to 35.7% in the fourth resource fair.

In response to expressed community need, the ICTR and CEAL team developed an online repository that allowed local CBOs, faith-based organizations, and other community associations to review 16 brochures of vetted COVID-19 health information. Viewers were also able to request up to 200 copies, in English or Spanish to distribute to their community [19].

After the first resource fair, the DT changed its approach to conducting community resource fairs. Through weekly meetings and discussions with BC partners, it became evident that there was a large number of resource fairs being conducted by different organizations. Therefore, the team transitioned from conducting BC-organized resource fairs to increasing the capacity of BC member organizations to conduct their own fairs.

The DT was resilient and troubleshooted to deal with implementation issues. For example, when there was no power supply to run the sound system at the second resource fair, and quickly ordered a generator and was ultimately able to activate the sound system.

Each member of the DT utilized their skill set to contribute to the planning and execution of the resource fairs. The DT co-chair maintained constant communication with staff from Baltimore City. Using these relationships helped to expedite the process of obtaining needed permits. Another DT member had connected with local city leadership and ensured that elected officials were present in person on the day of the events. Another DT member designed and produced promotional materials.

Implementation of the fairs was facilitated by open and transparent communication around the delegation of tasks around each person’s strengths. The resource fairs also provided an opportunity for community members to develop their skills in a safe and supportive space. One DT member

emphasized:

“*It had become clear to me that I need to utilize my skills/talent, if I wanted to be a productive member of this organization… I began to find my place as a resourceful individual, going to suggested sites that I was considered and returning with information/photos of this or that site. [This included] bringing in people who could help us as a team achieve our goal, finding places to store our equipment when needed. Coming early to set up equipment and helping out wherever I could or asked to*.”

In the planning process, another CBO member noted that:

“*Community providers get meaningful discussions*.” - CBO member

Innovative strategies were used for funding and organizing events, especially around flexibility in budget allocation. For each resource fair, the DT received block funding from ICTR ranging from $3000 to $5000. By providing block funding, the committee was allowed flexibility in allocating resources. The funding could be spread across a variety of items, and the DT determined the funding for each specific line item.

For example, this included funding for food, activities for children (e.g., a moonbounce), DJ, and portapotties. The team was then able to reach out to other organizations to secure additional smaller sponsorships for items such as youth volunteer stipends and covering food costs.

## Discussion

Collaboration between CEnR teams and CBOs is instrumental in driving the success of community resource fairs. These partnerships, characterized by a wide range of expertise, resources, and connections, can amplify the impact of these events. The strong community ties of CBOs enable them to recognize and address local needs, pool their collective knowledge and networks to create a comprehensive service continuum and provide a more inclusive and culturally relevant approach, ensuring that the resource fairs cater to the diverse needs of the community. This collaboration ensures effective outreach, resulting in greater community participation and engagement. A shared commitment to community well-being underscores the significance of these partnerships, leading to success of community resource fairs.

This case study provides important lessons that can increase the effectiveness of other community-academic partnerships. To date, CEnR teams have primarily focused on community partnerships in the context of research activities. In the partnership described, sustained engagement with community planners and academic partners to implement health fairs built trusting relationships, allowing the CEnR teams to learn from the community about optimal research participation. CBOs were able to leverage institutional resources and increase their capacity by obtaining student volunteer support, financial resources, and development and production of promotional materials. The deep roots that CBOs have within the community allowed them to rapidly and effectively identify and address specific needs and disseminate reliable information. Community resource fairs are an especially useful resource to reach out to community members and reach neighborhoods where they are based. It is a low-pressure area to provide resources to address SDoH like housing, food, utilities, and financial resources while also connecting families to health information and health services. Often, CBOs collect contact information and share application forms with attendees to allow them to follow up and potentially continue an engagement after the resource fair. Additionally, hosting resource fairs in different locations allows CBOs to expand their reach into other underserved zip codes.

*“In coming to JH in Baltimore in 2011 I recognized the community and university were unique in their history, talents, and collaborations. Getting to know the community was vital to my patient service, teaching of genetics and public health, and research.”* - Academic researcher

Consistent with CEnR literature, this study found that building trust and maintaining an open channel of communication were essential to planning and implementation [9]. In 2.5 years, community members who organized and interacted with the resource fair stayed consistent over time. The continuity over time that members volunteered demonstrates trust held between BC and the community. Open communication and transparency at each level of planning and implementation allowed members to actively engage in this iterative process. This promoted equitable contributions, strengthening community trust in research, which are important practices for Clinical and Translational Science Research teams [20].

Our case study highlights the reach of resource fairs when implemented with a community-centered lens. As an effective intervention for addressing SDoH, well planned fairs provide resources across a vast array of areas (e.g., food, housing, financial, health, education, youth, family) [3]. Implementation through community mobilization can achieve community and system-level changes [8,10].

The community health resource fairs specifically considered structural barriers to address SDoH. We addressed geographic barriers by implementing the fairs in different neighborhoods that would be accessible by foot or public transportation. The resource fairs were always free for community members to participate in, eliminating economic barriers. DT members did outreach to promote the fairs by going door-to-door to hand out flyers in order to decrease technological barriers. Additionally, the fair locations were also handicapped accessible.

### Limitations

This study had several limitations. This case description was not designed as a research study, and data collection was incomplete for the fifth resource fair. This case relied on vendor surveys and counts of individuals who visited vendor tables as engagement metrics, and responses may have been subject to recall bias. No demographic data, such as zip code were collected, limiting this study’s ability to understand whether the intervention was adequately reaching individuals from underserved areas. Given participant hesitancy to provide detailed demographic information, this case only captured estimates of the number of attendees and the vendor services they visited during the event.

### Implications

This iterative approach to building trusting relationships led to a mutually beneficial partnership between CEnR teams and CBOs, opening an opportunity to shift the paradigm for how CEnR teams can promote sustained engagement with communities. Modifying CEnR activities to include support for community-led planning can build trust and create more sustainable partnerships. This is critical since CBOs interface directly with members of the community and can provide the best insights for organizing events that are inclusive and effectively address community needs.

It can be difficult for CEnR teams and CBO partners to establish rules of engagement and carry out coordinated activities. One structural barrier is the onerous academic mechanisms for funding and a research budget’s lack of flexibility to allow for funding to adapt to evolving community-identified needs, including provision of stipends to individuals who are not research participants. Additional funding was required from other external funders to sponsor specific elements of the resource fair budget, such as stipends for youth volunteers, promotional flyers, and food. For the resource fairs described in this study, we relied in part on funds provided directly from Baltimore CONNECT, which were subject to different constraints than a university.

Another opportunity for growth is to use the fairs as opportunities for targeted discussions. Bringing together groups of community members provides opportunities to help them voice their concerns and interface with city officials and policymakers. Future resource fairs could aim to have structured forums for community members to meet with leaders to voice their needs and priorities. Forums also provide opportunities for community members to voice research priorities that are important for their communities.

The collaborations, resource sharing, and skills development that occur in workgroups like the DT can also expand beyond the committee’s goals. Team members have reported that they were able to use the skills for resource fair planning, requests for funding, and other community-centered activities. CEnR teams were also asked for additional health information support from vendors outside of the resource fairs, allowing them to expand their impact.

*As Community Advocate living in East Baltimore… my experiences have prepared me to bridge gaps and cultivate meaningful relationships and carry that knowledge over to educate and improve the quality of life for others. Through trial and error, I’ve found it to be critical and utmostly respectful to engage community at all levels of planning. - Community leader*

**Figure 4.**
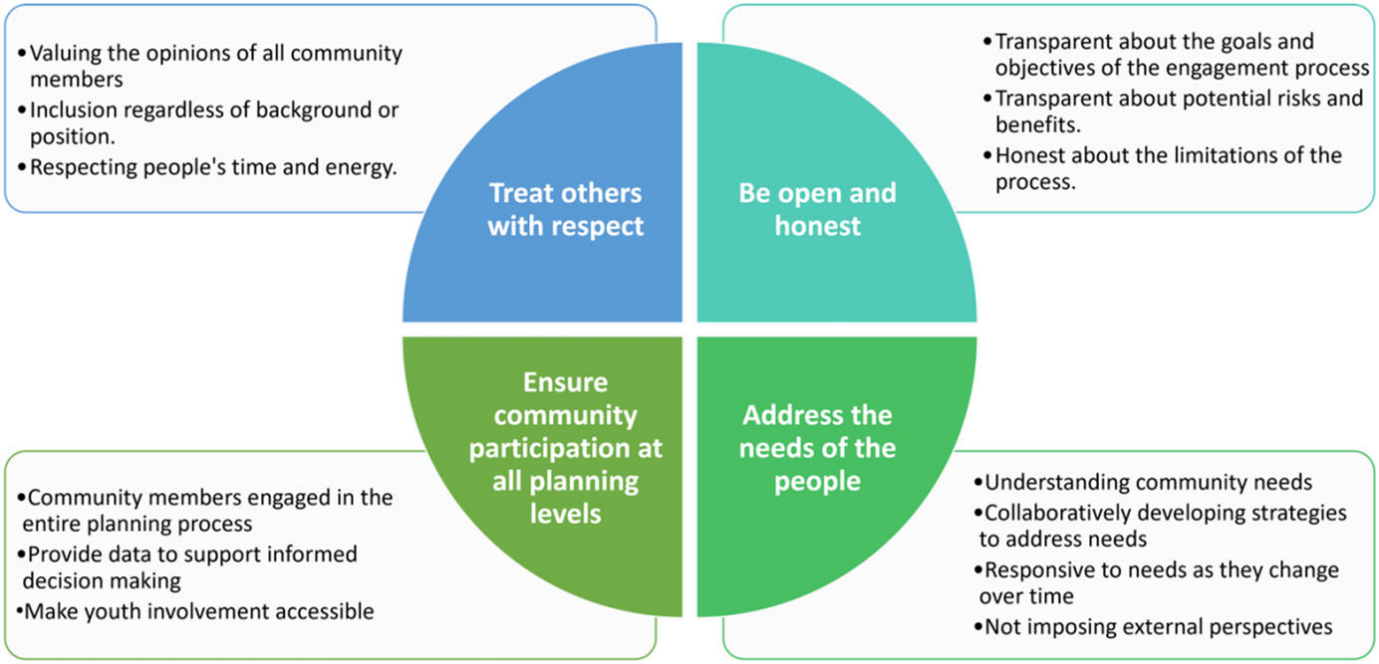
Principles for community engagement as developed by dream team members. By following these key principles, Community-Engaged Research practitioners can increase the likelihood of achieving successful outcomes.

The participants involved in this case study generated principles and practices to guide true collaboration with members of underserved communities (shown in Figure 4)

## Conclusions

The collaborative efforts demonstrated by the BC Dream team exemplify a successful model of community engagement and resource and information sharing. The ongoing challenges and barriers encountered during the past two and a half years of collaboration highlight the need for continuous improvement and adaptation in community-driven initiatives. In the words of some of our community collaborators:

“*As a community advocate for over 27 years this group has set precedent for other efforts to come. The work each agency has done and will continue to do throughout Maryland has made a positive impact on its underserved communities*.” - DT Committee member

The DT was an effective convening organization that helped CBOs and CEnR teams to unite around one common goal and let the community care about them. In working together, these organizations provide a more comprehensive range of services to residents of Baltimore City. For example, the CBOs represented within DT provide access to mental health services, job information, and COVID-19 information and vaccinations, while other CBOs invited as resource fair vendors provide a variety of other services, such as food, clothing, and housing assistance.

“*This collaboration has helped to improve the quality of life for many residents of Baltimore City*.” - CBO member
